# *Mycoplasma hominis* impacts gene expression in *Trichomonas vaginalis*

**DOI:** 10.1007/s00436-018-5761-6

**Published:** 2018-01-24

**Authors:** Ursula Fürnkranz, Birgit Henrich, Julia Walochnik

**Affiliations:** 1 Institute of Specific Prophylaxis and Tropical Medicine, Centre for Pathophysiology, Infectiology and Immunology, Medical University of Vienna, Kinderspitalgasse 15, A-1090 Vienna, Austria; 2 Institute of Medical Microbiology and Hospital Hygiene, Heinrich Heine University, Düsseldorf, Germany

**Keywords:** Symbiosis, *Trichomonas vaginalis*, *Mycoplasma hominis*, mRNA, PFOR

## Abstract

In Europe, up to 90% of isolated *Trichomonas vaginalis* strains are naturally infected with *Mycoplasma hominis*, a facultative pathogen of the human genital tract. The consequences of this endosymbiosis are not yet well understood. The aim of the current study was to evaluate the impact of natural and artificial infections with *M. hominis* on the RNA expression levels of metronidazole susceptibility-associated genes of *T. vaginalis.* Three *T. vaginalis* strains (TVSS10−, TVSS25−, G3) without *M. hominis*, as well as the same strains naturally (TVSS10+, TVSS25+) and artificially (G3-MhSS25, TVSS25-MhSS25) infected with *M. hominis*, were investigated for their expression profiles of three genes associated with metronidazole resistance (ferredoxin, flavin reductase 1 and pyruvate:ferredoxin oxidoreductase). The minimal inhibitory concentrations (MICs) of metronidazole were evaluated for all combinations and the respective *M. hominis*-free *T. vaginalis* strains were used as controls. The sole presence of *M. hominis* led to a down-regulation of metronidazole susceptibility-associated genes in all *T. vaginalis* strains tested. Interestingly, the effect was more prominent in the artificial symbioses. Moreover, a twofold enhancement of metronidazole tolerability was observed in three infected *T. vaginalis* strains, compared to the respective strains without *M. hominis*. In conclusion, *M. hominis* had an impact on gene expression in all *T. vaginalis* strains and on metronidazole MIC in all but one strain tested.

## Introduction

*Trichomonas vaginalis* is the causative agent of trichomonosis, the most common non-viral sexually transmitted disease worldwide. *T. vaginalis* has been shown to undergo symbiosis with the bacterium *Mycoplasma hominis*, commonly found in the vaginal flora of asymptomatic women (Taylor-Robinson 2017), but also causing symptomatic infections (Hartmann 2009). The symbiosis of *T. vaginalis* with *M. hominis* has been observed in up to 94% of clinical isolates of *T. vaginalis*, e.g. in patients from Italy and Africa (Rappelli et al. 1998). A few aspects of this symbiosis, mainly concerning the pathogenicity, immunology and metabolism of *T. vaginalis*, have already been investigated (Fichorova et al. 2017), including also the impact of *M. hominis* on the metronidazole resistance of naturally infected clinical isolates of *T. vaginalis*. One of these studies revealed a correlation between the presence of *M. hominis* and resistance (Xiao et al**.** 2006); two other studies did not (Butler et al. 2010; da Luz Becker et al. 2015). Resistance of *T. vaginalis* against metronidazole has been associated with a down-regulation of metronidazole-activating enzymes, particularly pyruvate:ferredoxin oxidoreductase (PFOR) and ferredoxin (Land et al. 2001). Another form of resistance is caused by decreased oxygen scavenging capacity being responsible for higher oxygen (O_2_) concentrations inside the hydrogenosomes. Oxygen can then re-oxidise the nitroradical anions, thus decreasing their effect. Flavin reductase is part of the oxygen defence system in *T. vaginalis* and has been reported to be not expressed in highly resistant *T. vaginalis* (Leitsch et al. 2014). To date, little is known on possible mechanisms of how *M. hominis* may interact with *T. vaginalis*. As reported recently, bacteria inside eukaryotic cells can influence their host to enhance their own chances for intracellular survival. *Legionella pneumophila* for example impairs the cell cycle of its host, *Acanthamoeba castellanii*, by decreasing specific host mRNA levels (Mengue et al. 2016). Another symbiont of *T. vaginalis*, the double-stranded RNA *Trichomonas vaginalis* virus (TVV) has been observed very often in combination with the presence of *M. hominis* (da Luz Becker et al. 2015). Moreover, the presence of the virus has been associated with metronidazole susceptibility (Snipes et al. 2000; Malla et al. 2011).

The aim of the present study was to assess the impact of *M. hominis* on RNA expression in *T. vaginalis* by screening genes related to metronidazole susceptibility. This model was chosen because it is well established and the effects of possibly observed changes in RNA expression can easily be compared to phenotypic characteristics by performing minimal inhibitory concentration (MIC) assays. The multiplicity of infection (MOI) in all assays was investigated to report influences of different counts of bacteria on RNA expression and/or metronidazole susceptibility. Moreover, we screened the *T. vaginalis* strains used for the presence of the *T. vaginalis* virus (TVV).

## Material and methods

### Strains

Three strains of *T. vaginalis* were used: the mycoplasma*-*free strain *T. vaginalis* G3 (type strain, ATCC_Prae-98) and the naturally *M. hominis*-infected strains TVSS25+ and TVSS10+ (kindly provided by Prof. Fiori, University of Sassari). All *T. vaginalis* strains were cultivated at 37 °C in 12-cm^2^ flasks completely filled with TYM (trypticase–yeast–maltose) medium (Diamond 1957). In order to clear TVSS25+ and TVSS10+ from *M. hominis*, TYM was supplemented with Plasmocin™ (Invivogen), final concentration of 25 μg/ml according to the manufacturer’s protocol. TVSS25+ and TVSS10+ were sub-cultured every second day in this medium for 2 weeks and the absence of *M. hominis* was confirmed by incubation of the two strains (further referred to as TVSS25− and TVSS10−) in *Mycoplasma* medium PPLOa (Lin and Kass 1975) to screen for growth of *M. hominis*. Furthermore, qPCR on the *p80*-gene (fw 5′-TTGAGGCACAGCAATAGC-3′; rw 5′-AAGGCTTAGGTAAGGAATGATTAG-3′) of *M. hominis* was performed to verify its absence. TVSS25− and TVSS10− were cultured mycoplasma-free for more than 6 months before being used for drug screening and artificial infection in the case of TVSS25−.

*M. hominis* strain MhSS25 was used to infect *T. vaginalis* G3 and TVSS25−. MhSS25 was isolated from TVSS25+ by transferring 1 ml of TVSS25+ into PPLOa medium in which *T. vaginalis* is not able to survive. After 48 h of incubation in PPLOa at 37 °C, a colour change from orange to red indicated growth of *M. hominis* which was subsequently aliquoted and frozen for later use.

### Screening for TVV

Identification of TVV was performed according to the protocol of Fraga et al. (2005). In short, whole genomic DNA of all *T. vaginalis* strains available in our laboratory including the three isolates used in the current study was separated by gel electrophoresis using a 1% agarose gel. A visible band between 4.3 and 4.8 kb confirmed the presence of TVV.

### Artificial co-culture

Co-cultures of *T. vaginalis* and *M. hominis* were set up as described by Morada et al. (2010) with a few modifications: 100 μl of *M. hominis* culture, consisting of 10^8^–10^9^ colour changing units (CCU) *M. hominis*/ml was added daily to 2 ml of an exponentially growing *T. vaginalis* culture (10^4^ cells/ml on the first day of infection) for 4 days in TYM at 37 °C. After 4 days, the respective co-culture was transferred to a 12-cm^2^ flask and further cultivated up to 35 days by passaging every second day. The presence of *M. hominis* was verified by transferring 1 ml of the respective co-culture to PPLOa medium and qPCR. Viability of *T. vaginalis* was determined microscopically, non-motile *T. vaginalis* were considered dead.

### RNA extraction, cDNA synthesis and qPCR

Total RNA of *T. vaginalis* with and without *M. hominis* was extracted and converted to cDNA. RNA extraction was performed between days 8 and 30 of culture without metronidazole challenge. One flask (35 ml culture consisting of 1–5 × 10^5^ cells/ml) was centrifuged at 600×*g* for 10 min, washed with PBS and the cell sediment was resuspended in 600 μl lysis buffer RLT (RNeasy Kit; Qiagen, Hilden, Germany). RNA was subsequently prepared according to the manufacturer’s protocol. To digest traces of DNA, all RNA samples were treated with DNase I (Roche Molecular Biochemicals) according to a slightly modified protocol of Henrich et al. (1999). In brief, 1 μl DNase I (10,000 U/ml) was added to 9 μg RNA in 40 μl RNase-free water and incubated at 37 °C for 20 min, followed by 25 °C for 40 min and 70 °C for 5 min. After RNA precipitation at − 80 °C, RNA was resolved, photometrically measured and 1 μg of total RNA was converted to random primed cDNA according to the manufacturer’s instructions (Invitrogen, Life Technologies). To screen for possible genomic DNA residues in the RNA, 1 μg of RNA was subjected to qPCR, too. qPCR assays were carried out in a total volume of 25 μl, consisting of 2× MesaGreen MasterMix (Eurogentec), 300 nM of each primer and 2.5 μl of cDNA using the following protocol: starting with 50 °C for 10 min and 95 °C for 10 min, followed by 45 repeats of 15 s at 95 °C and 1 min at 60 °C, and finalised by melting point analysis. Primers for *T. vaginalis* genes associated with metronidazole resistance are listed in Table 1; β-tubulin was used as a reference. For all primers used, gradient qPCRs were run to evaluate optimal annealing temperatures. All qPCRs performed well at an annealing temperature of 60 °C (data not shown).

**Table 1:** Sequences and references of the primers used in this study

Gene for	Forward primer (5′–3′)	Reverse primer (5′–3′)
Flavin reductase 1	CTTGATGTCTCACATGCACG	TTGGCTGAATCAGCGAAACG
Reference: Leitsch et al. (2014)		
PFOR b	CTGCAAGCTCCTTACACAGC	AAGAGGGAGTTAGCCCAAGC
Reference: Mead et al. (2006)		
Ferredoxin	TGCCGCTTTGGAACAATCA	TGTCTGGTCATCTTCGAACTTGA
In house design by B. Henrich		
β-Tubulin	AAGATGGGTGTTTTAAGCTAGATAAGT	CGTCTTCAAGTATGCCCCAGTAC
Reference: Schirm et al. (2007)		

### Drug screening

Metronidazole-susceptibility testing of natural and artificial symbioses as well as *T. vaginalis* without *M. hominis* was performed between days 8 and 35 of culture. Drug screening was performed as established by Leitsch et al. (2012). Metronidazole (Sigma; M3761) was stored as a stock solution of 10 mg/ml DMSO and diluted to 1024 μg/ml in TYM at the day of the experiment. In short, 150 μl TYM with descending metronidazole concentrations (1024 to 1 μg/ml, 1:1 diluted in TYM) was incubated with 150 μl of 1–3 × 10^5^ *T. vaginalis* cells/ml (± *M. hominis*) in exponential growth phase under aerobic conditions in 96-well microtitre plates resulting in 512 to 0.5 μg/ml final concentration metronidazole. Viability was checked microscopically before the tests and only clones with more than 90% motile cells were used. After 48 h of incubation at 37 °C, cell viability was examined microscopically. The concentration at which no motile *T. vaginalis* cell was observed was defined as minimal inhibitory concentration (MIC). Untreated cultures of the respective strain, incubated in the same plate, were used as controls.

### Multiplicity of infection (MOI)

Genomic DNA was extracted on the same day of the respective metronidazole susceptibility or RNA extraction assay by proteinase K digestion. In brief, 1 ml of culture (10^5^ to 10^6^ *T. vaginalis* cells/ml) was centrifuged at 600×*g* for 10 min, thus pelleting only *T. vaginalis* with attached and intracellular *M. hominis* but not planktonic *M. hominis* cells. The cell pellet was resuspended in 150 μl proteinase K solution (final concentration of 66.6 μg proteinase K in 10 mM Tris–HCl pH 8.0), incubated at 56 °C for 50 min and inactivated by 94 °C for 20 min. The ratio of the two pathogens was measured by qPCR based on quantification of *M. hominis (p80)* and *T. vaginalis* (*L23861*; Schirm et al. 2007) genomic equivalents. MOI was calculated as genomic equivalents of *M. hominis*/genomic equivalents of *T. vaginalis.* The qPCR protocol was the same as described in the RNA extraction section, using 2.5 μl genomic DNA and shortened to 35 cycles.

### Statistics

RNA expression levels are represented as the mean results of *n* individual experiments ± mean deviation, as well as percent up- or down-regulation. Metronidazole susceptibilities are expressed as the mean including minimal and maximal MIC values measured of *n* experiments set up in duplicates. Student’s *t* test was used to calculate *p* values; *p* values of < 0.05 were considered statistically significant. Pearson correlation coefficient *r* was calculated using http://www.alcula.com/calculators/statistics/correlation-coefficient.

## Results

This is the first study comparing the RNA expression levels of metronidazole susceptibility-associated genes and the respective MICs in *T. vaginalis* strains naturally or artificially infected with *M. hominis* and their *M. hominis*-free clones.

### RNA expression

**Fig. 1:**
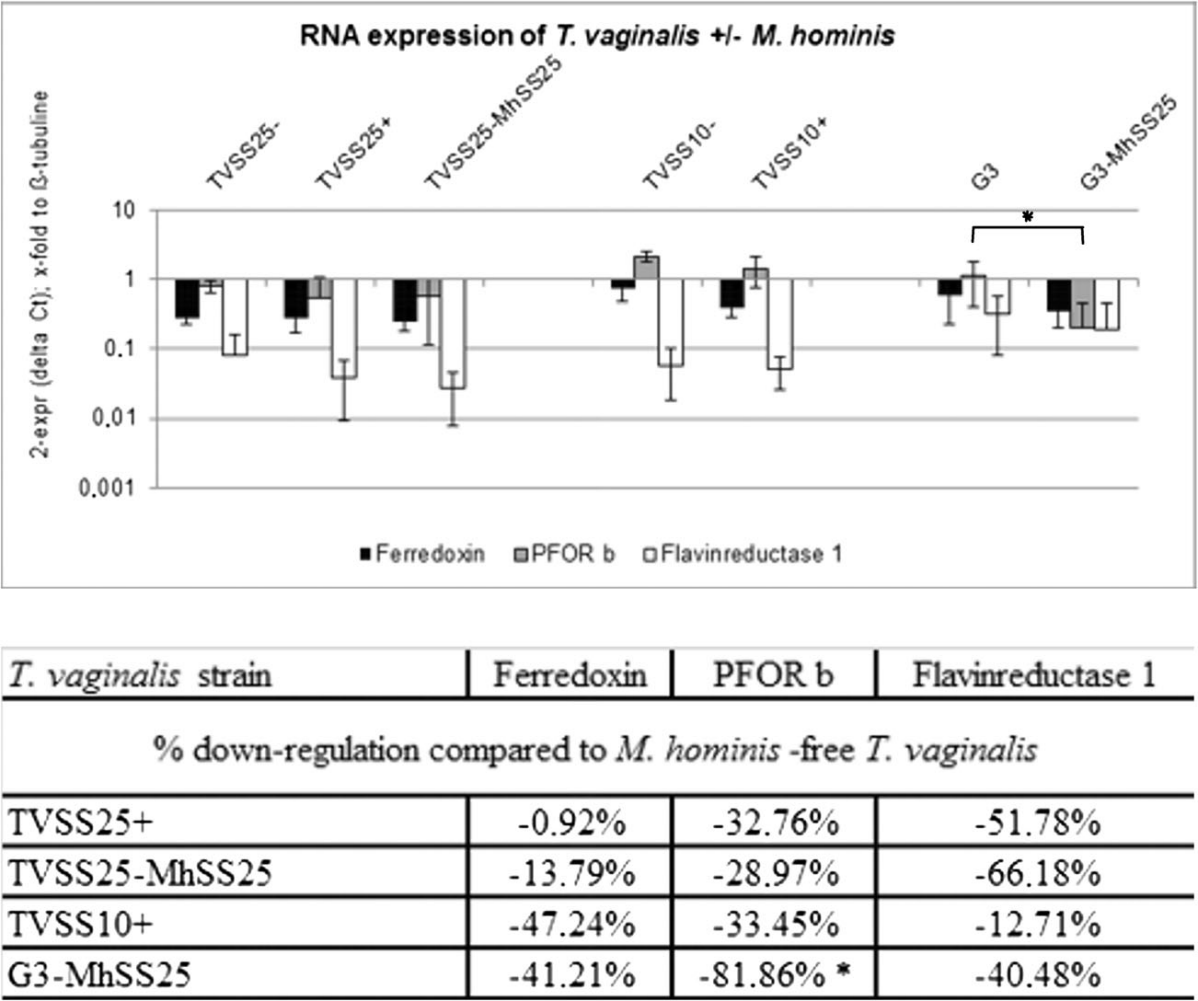
RNA expression levels are depicted as mean ± mean deviation of three (TVSS10−, TVSS10+) and four (TVSS25−, TVSS25+, TVSS25-MhSS25, G3, G3-MhSS25) independent experiments. Statistically significant differences (*p* < 0.05) in expression levels between *M. hominis*-infected and -uninfected *T. vaginalis* are labelled with an asterisk (*). Down-regulation of mRNA expression levels for ferredoxin, PFOR b and Flavin reductase 1 in % was calculated as 100 − (ratio × 100). The ratio was calculated as 2^−ΔΔCt^ *M. hominis*-infected *T. vaginalis*/2^−ΔΔCt^ *T. vaginalis*

The RNA-expression profiles of all *T. vaginalis* with and without *M. hominis* for ferredoxin, PFOR b and flavin reductase 1 are shown in Fig. 1. Controls of residue DNA in the RNA samples showed at least 10 times lower Ct values than RNA samples (data not shown), corresponding to less than 0.1% residual genomic DNA. Different MOIs and time periods of culturing had no influence on the RNA expression levels of the genes investigated (data not shown). All RNA-expression levels were down-regulated when *M. hominis* was present in all symbioses tested. This effect was less prominent in TVSS25+ and more striking in the other symbioses investigated. Furthermore, the down-regulation was more outstanding in the artificial co-culture G3-MhSS25 (> minus 40% for all genes investigated). PFOR b was the gene most affected in all symbioses and was statistically significantly (*p* = 0.005) down-regulated in G3-MhSS25 compared to the parental *M. hominis*-free G3.

### Drug screening

**Fig. 2:**
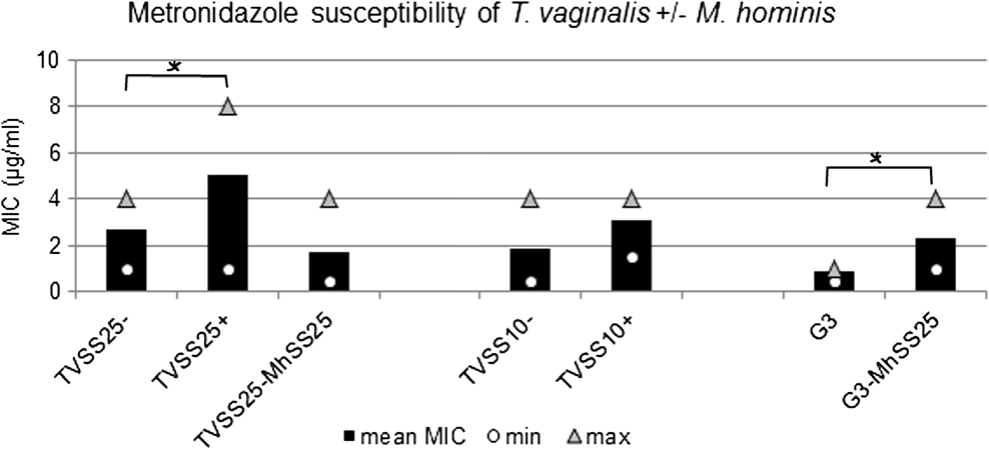
Metronidazole susceptibility of *M. hominis*-free *T. vaginalis* is depicted as mean MIC of three (G3) and four (TVSS25−, TVSS10−) experiments set up in duplicate. The results of the *M. hominis*-infected TVSS25+, TVSS10+, G3-MhSS25 and TVSS25-MhSS25 are shown as mean of six to nine experiments in duplicate. Minimum (○) and maximum (Δ) MICs observed are included. Statistically significant differences are marked by an asterisk (*)

When comparing the *M. hominis*-infected *T. vaginalis* to the respective mycoplasma-free *T. vaginalis*, a twofold increase of metronidazole MIC was observed, except in TVSS25-MhSS25 (Fig. 2). In the case of TVSS25+, enhanced tolerability to metronidazole compared to TVSS25− was seen when elevated MOIs (more bacteria present) were observed (data not shown). Pearson correlation coefficient calculation (*r* = 0.43) revealed a positive correlation between higher MOI (≥ 1) and enhanced metronidazole tolerability in the natural *M. hominis*-infected TVSS25+. Furthermore, the twofold enhancement of metronidazole MIC of TVSS25+ was statistically significant (*p* = 0.001). G3-MhSS25 showed a statistically significantly enhanced MIC to metronidazole compared to G3 alone (*p* < 0.001), irrespective of the MOI. TVSS10+ also showed higher, although not statistically significant (*p* = 0.07), MIC irrespective of the MOI. The artificial symbiosis of TVSS25-MhSS25 did not reveal higher metronidazole MIC than the *M. hominis*-cleared TVSS25−.

### Presence of TVV

**Fig. 3:**
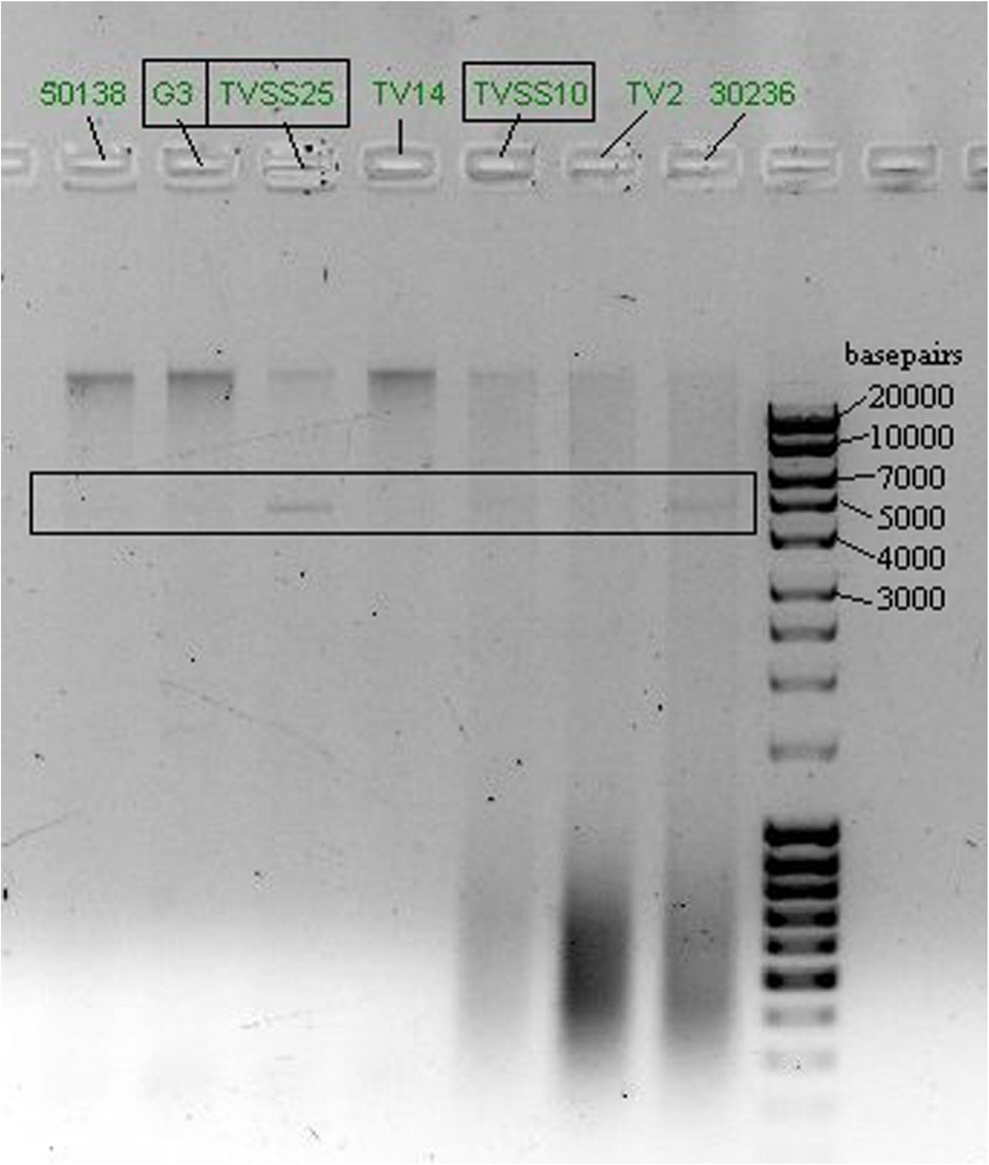
Gel-electrophoresis picture for the proof of the presence of TVV; whole genomic DNA from seven *T. vaginalis* isolates available in our laboratory was separated. The three isolates investigated in the current study are marked with squares. The area of suspected presence of TVV between 4 and 5 kb is marked with a square

TVSS25− and TVSS10− were TVV-positive. TVSS25− showed a strong band at ~ 5 kb (Fig. 3), TVSS10− showed a faint band. G3 showed no band and was considered TVV-free.

## Discussion

In this study, it was shown for the first time that *M. hominis* impacts the gene expression of metronidazole resistance-associated genes in *T. vaginalis.* Moreover, this is the first study directly comparing natural and artificial symbioses.

*M. hominis* has already been shown to impact the gene expression of infected HeLa cells (Hopfe et al. 2013). Until now, the effects of *M. hominis* on *T. vaginalis* have been mostly investigated phenotypically, e.g. *M. hominis* enhancing ATP production, growth rate and haemolytic activity of *T. vaginalis* (Margarita et al. 2016), as well as altering the immune response of host cells to the infection (Mercer et al. 2016; Fiori et al. 2013). However, the influence of *M. hominis* on mRNA levels in *T. vaginalis* has not been investigated in depth so far. Morada et al. (2010) investigated mRNA expression levels of three arginine deiminase (ADI) genes in a *T. vaginalis* strain artificially infected with *M. hominis* and the *M. hominis*-free counterparts. They revealed that despite elevated ADI activity in the *M. hominis*-infected *T. vaginalis* strain, two of the genes were down-regulated and only one was up-regulated by 30% in the presence of *M. hominis*. Thus, they concluded that *M. hominis* had no effect on the mRNA expression of these genes. In the present study, we observed a down-regulation of between 1 and 66% in the naturally infected *T. vaginalis* strains (TVSS25+, TVSS10+) and this down-regulation was even more pronounced (13.7% to 82%) in the artificially infected ones (G3-MhSS25, TVSS25-MhSS25) compared to the respective *M. hominis*-free clones. Importantly, the data in the present study were obtained without metronidazole challenge of the *T. vaginalis* strains, thus the observed down-regulation of the genes investigated is not in response to metronidazole itself. In a recent study, *Bacteroides fragilis* RNA-expression patterns were investigated before and after treating the bacteria with sub-lethal concentrations of metronidazole (de Freitas et al. 2016). The authors found no common genes expressed in non-treated bacteria and those under drug pressure. Thus, they suggested that with metronidazole challenge other genes are expressed than without. da Luz Becker et al. (2015) tested naturally infected and *M. hominis*-free clinical isolates of *T. vaginalis* for their metronidazole resistance and investigated the mRNA levels of PFOR. Two *T. vaginalis* strains tested showed the same resistance to metronidazole (36.5 μg/ml), although one had highly up-regulated PFOR levels whereas the other had down-regulated PFOR levels. The *T. vaginalis* strain with up-regulated PFOR was negative for *M. hominis*, whereas the strain with down-regulated PFOR was naturally infected with *M. hominis*. It has already been shown that PFOR-deficient *T. vaginalis* may still be rather susceptible to metronidazole (Rasoloson et al. 2002). In our study, PFOR b was down-regulated in all pairs of strains tested (statistically significantly for G3-MhSS25) compared to the respective *M. hominis*-free *T. vaginalis*. Thus, it can be speculated that, in this context, down-regulated PFOR does not necessarily indicate a higher MIC to metronidazole, but simply the presence of *M. hominis*. Further studies will be needed to clarify this effect of *M. hominis* on *T. vaginalis*.

Nevertheless, in our study the *M. hominis*-infected *T. vaginalis* strains showed higher MIC to metronidazole than the respective non-infected strains. This was statistically significant in the case of the naturally infected TVSS25+ and artificially infected G3-MhSS25. This is the first time that the same *T. vaginalis* strains artificially infected and *M. hominis*-free were investigated for their metronidazole susceptibilities. Previous studies only investigated naturally infected *T. vaginalis* for their metronidazole susceptibilities (Xiao et al. 2006; Butler et al. 2010; da Luz Becker et al. 2015). In our study, the *M. hominis*-cleared TVSS25− and TVSS10− clones showed lower MIC to metronidazole than the parental naturally infected TVSS25+ and TVSS10+ clones. This differs from the findings of Butler et al. (2010) who did not observe changes in the metronidazole susceptibility when clearing naturally infected *T. vaginalis* from *M. hominis*. The difference might be explained by the fact that in the present study the *M. hominis*-cleared TVSS25− and TVSS10− were cultured *M. hominis*-free for more than 6 months before assaying metronidazole susceptibility, while Butler et al. (2010) used *T. vaginalis* cleared of *M. hominis* after 4 weeks of treatment with Plasmocin. The parental strains TVSS25+ and TVSS10+ were kept in culture for half a year as well before the metronidazole susceptibility assays were performed. In the case of the naturally infected TVSS25+, the enhanced MIC was seen after longer cultivation periods (after 14 days) and elevated MOIs. As growth of *M. hominis* is not affected by metronidazole in vitro (Hartmann 2009 and own observations), it can be speculated that high densities of *M. hominis* decrease the final concentration of the drug. However, in the artificially infected G3-MhSS25 and the naturally infected TVSS10+, the metronidazole tolerability was enhanced independently of the MOI. Thus, the number of *M. hominis* cells cannot be the only decisive factor for an enhanced MIC to metronidazole. In no case was a true resistance induced, which is defined as tolerating higher concentrations than 50 μg metronidazole/ml (Butler et al. 2010). Unexpectedly, TVSS25-MhSS25—being re-infected with the same *M. hominis* strain it harboured before clearing—did not behave as the naturally infected strain (TVSS25+) concerning its metronidazole MIC. TVSS25-MhSS25 showed a comparable metronidazole susceptibility to its parental *M. hominis*-free TVSS25−. Nevertheless, the RNA expression profiles of TVSS25-MhSS25 resembled those of the naturally infected TVSS25+, where statistically significant enhanced tolerability was observed. Thus, more studies are needed to clarify if the down-regulation of the genes investigated at the mRNA level is more likely an evidence of the presence of *M. hominis* or of elevated MIC to metronidazole.

The presence of a double-stranded RNA virus TVV in *T. vaginalis* has been associated with metronidazole susceptibility in vitro (Snipes et al. 2000; Malla et al. 2011). In our study, two of the investigated isolates of *T. vaginalis* (TVSS25 and TVSS10) harboured TVV; all three isolates investigated were susceptible to metronidazole. However, as in our study, the TVV-free isolate G3 showed the lowest susceptibility to metronidazole and statistically significant enhancement of tolerability due to the presence of *M. hominis* occurred in one TVV-positive and one TVV-negative isolate; no correlation can be drawn between the presence of TVV and metronidazole susceptibility in our study. However, high presence of TVV has been observed in *T. vaginalis* isolates naturally harbouring *M. hominis* (da Luz Becker et al. 2015). Although the sample size of seven *T. vaginalis* isolates investigated in the present study is not meaningful, it was surprising to see that from the three TVV-positive *T. vaginalis* isolates, two naturally harboured *M. hominis*.

In conclusion, it can be stated that the presence of *M. hominis* affected the expression levels of the investigated *T. vaginalis* genes that have been described to all play a role in metronidazole resistance, especially PFOR. A high cell density of *M. hominis* in symbiosis is not sufficient for leading to an enhanced metronidazole MIC of *T. vaginalis* per se, as this was only observed in one pair of strains tested. To the best of our knowledge, this is the first study comparing different *T. vaginalis* strains that were artificially infected with the same strain of *M. hominis* and the first study comparing natural and artificial symbioses.

### Strengths and limitations of this study

#### Strengths

Two different strains of *T. vaginalis* in artificial and natural symbiosis with the same strain of *M. hominis* were compared. An additional naturally infected *T. vaginalis* strain was used as control. Three genes mostly described to be involved in metronidazole resistance were investigated.

#### Limitations

Other genes that have been described to be involved in metronidazole resistance have not been investigated.
